# 

**Published:** 1906-03

**Authors:** 


War on the Vampire; Laymen in the Lead.

VOL. XXI. MARCH, 1906. No. 9.

INDEXED
430

### TEXAS Medical Journal.

LIBRARY
SURGEON GENERAL'S OFFICE
MAR. 13-1906

SUBSCRIPTION, $1.00 A YEAR, IN ADVANCE.

SINGLE COPIES, 10 CENTS.

PUBLISHED AT AUSTIN, TEXAS, BY

F. E. DANIEL, M. D.,

PROPRIETOR.

PRINTED BY THE VON BOECKMANN-JONES CO.

Entered at the Postoffice at Austin, Texas, as Second-class Mail Matter.

